# Assessing ChatGPT’s Mastery of Bloom’s Taxonomy Using Psychosomatic Medicine Exam Questions: Mixed-Methods Study

**DOI:** 10.2196/52113

**Published:** 2024-01-23

**Authors:** Anne Herrmann-Werner, Teresa Festl-Wietek, Friederike Holderried, Lea Herschbach, Jan Griewatz, Ken Masters, Stephan Zipfel, Moritz Mahling

**Affiliations:** 1 Tübingen Institute for Medical Education Faculty of Medicine University of Tübingen Tübingen Germany; 2 Department of Psychosomatic Medicine and Psychotherapy University Hospital Tübingen Tübingen Germany; 3 University Department of Anesthesiology and Intensive Care Medicine University Hospital Tübingen Tübingen Germany; 4 Medical Education and Informatics Department College of Medicine and Health Sciences Sultan Qaboos University Muscat Oman; 5 Department of Diabetology, Endocrinology, Nephrology Section of Nephrology and Hypertension University Hospital Tübingen Tübingen Germany

**Keywords:** answer, artificial intelligence, assessment, Bloom’s taxonomy, ChatGPT, classification, error, exam, examination, generative, GPT-4, Generative Pre-trained Transformer 4, language model, learning outcome, LLM, MCQ, medical education, medical exam, multiple-choice question, natural language processing, NLP, psychosomatic, question, response, taxonomy

## Abstract

**Background:**

Large language models such as GPT-4 (Generative Pre-trained Transformer 4) are being increasingly used in medicine and medical education. However, these models are prone to “hallucinations” (ie, outputs that seem convincing while being factually incorrect). It is currently unknown how these errors by large language models relate to the different cognitive levels defined in Bloom’s taxonomy.

**Objective:**

This study aims to explore how GPT-4 performs in terms of Bloom’s taxonomy using psychosomatic medicine exam questions.

**Methods:**

We used a large data set of psychosomatic medicine multiple-choice questions (N=307) with real-world results derived from medical school exams. GPT-4 answered the multiple-choice questions using 2 distinct prompt versions: detailed and short. The answers were analyzed using a quantitative approach and a qualitative approach. Focusing on incorrectly answered questions, we categorized reasoning errors according to the hierarchical framework of Bloom’s taxonomy.

**Results:**

GPT-4’s performance in answering exam questions yielded a high success rate: 93% (284/307) for the detailed prompt and 91% (278/307) for the short prompt. Questions answered correctly by GPT-4 had a statistically significant higher difficulty than questions answered incorrectly (*P*=.002 for the detailed prompt and *P*<.001 for the short prompt). Independent of the prompt, GPT-4’s lowest exam performance was 78.9% (15/19), thereby always surpassing the “pass” threshold. Our qualitative analysis of incorrect answers, based on Bloom’s taxonomy, showed that errors were primarily in the “remember” (29/68) and “understand” (23/68) cognitive levels; specific issues arose in recalling details, understanding conceptual relationships, and adhering to standardized guidelines.

**Conclusions:**

GPT-4 demonstrated a remarkable success rate when confronted with psychosomatic medicine multiple-choice exam questions, aligning with previous findings. When evaluated through Bloom’s taxonomy, our data revealed that GPT-4 occasionally ignored specific facts (remember), provided illogical reasoning (understand), or failed to apply concepts to a new situation (apply). These errors, which were confidently presented, could be attributed to inherent model biases and the tendency to generate outputs that maximize likelihood.

## Introduction

The recent developments in artificial intelligence (AI) have transformative potential for various fields, including medicine [1] and medical education [2]. In November 2022, OpenAI launched GPT-3 (Generative Pre-trained Transformer 3), a large language model (LLM) [3]. Its high-quality performance surprised even experts and generated huge public interest (particularly in school and higher education settings, where GPT-3 prompted manifold discussions on its potential benefits and harms) [4,5].

In medical education, LLMs have the potential to revolutionize current teaching approaches and thus ultimately improve physician performance and health care outcomes. However, before LLMs are thoroughly integrated into medical education, their performance in this context must be comprehensively assessed. It is especially important to evaluate the capabilities of AI and LLMs within educational theoretical frameworks.

One of the most-used frameworks in medical education is Bloom’s taxonomy [6,7] of learning outcomes, first introduced in 1956. Briefly, Bloom and subsequent colleagues developed a hierarchical classification of cognitive processes, ordered from lower-order cognitive skills—such as knowledge recall (remember) and comprehension (understand)—to higher-order thinking—such as application (apply), analysis (analyze), evaluation (evaluate), and creation (create) [8].

Since its first publication in 1956, this taxonomy has been used as a common language for educational instructors and still influences the field of medical education [8]. With his work, Bloom provided a significant contribution to what is now known as outcome-based education [9] and laid the foundation for other educational theories, such as Miller’s pyramid of clinical competencies [10,11]. While Bloom’s taxonomy is widely used and offers a structured approach to learning outcomes, some educators believe that its hierarchical nature might not always represent the complexity of learning [12].

Although derived from human learning processes, Bloom’s taxonomy provides an ideal framework to describe the cognitive processes that underlie success and failure. Recently, LLMs have been assessed for their (surprisingly mostly good) performance in various fields of medicine, ranging from specific subjects to board exams [13-15]. However, the errors made by LLMs have not been evaluated in detail. For example, while LLMs might successfully recall facts (remember), they might struggle to apply those facts to a different context, or vice versa. We acknowledge that applying terms such as “remember” and “struggle” are anthropomorphisms used for ease of reading, as an LLM currently does neither and merely generates responses based on language-usage statistical probabilities using a “next-word prediction paradigm” [16].

Therefore, we aimed to use Bloom’s taxonomy to gain a better understanding of the failures of LLMs. For human medicine education and the aforementioned use cases for LLMs, multiple-choice questions (MCQs) remain a primary written exam form and are used for summative and formative assessments [17]. In Bloom’s taxonomy, MCQs are often used to assess lower-order cognitive skills, such as knowledge recall (remember) and comprehension (understand), but they may also probe higher-order thinking, such as application (apply), analysis (analyze), and evaluation (evaluate) [18]. Thus, MCQs offer a suitable lens for evaluating different cognitive processes.

A medical field that relies heavily on language and factual understanding is instrumental to elucidating cognitive processes and correct or incorrect reasoning. Given its interplay of psychological, social, and biological factors, psychosomatic medicine offers such a case. The field’s heavy reliance on verbal and written communication for diagnosis and treatment makes it particularly challenging. Additionally, the combination of strict diagnostic criteria with a nuanced understanding of the patient’s language makes it an ideal testing ground for the capabilities of language models.

We present a mixed methods study designed to explore how GPT-4 performs in terms of Bloom’s taxonomy. First, we assessed the performance of GPT-4 with a large set of psychosomatic medicine exam questions and compared the results to responses from a cohort of medical students, thereby providing human comparison and quality indicators. For a deeper understanding of the results, we used qualitative methods to comprehend the model’s performance and to assess the strengths and weaknesses of LLMs in relation to Bloom’s taxonomy. The findings of this study provide critical insights into the practical applications and limitations of LLMs (such as GPT-4) in medical education.

## Methods

### Exams

**Figure 1:**
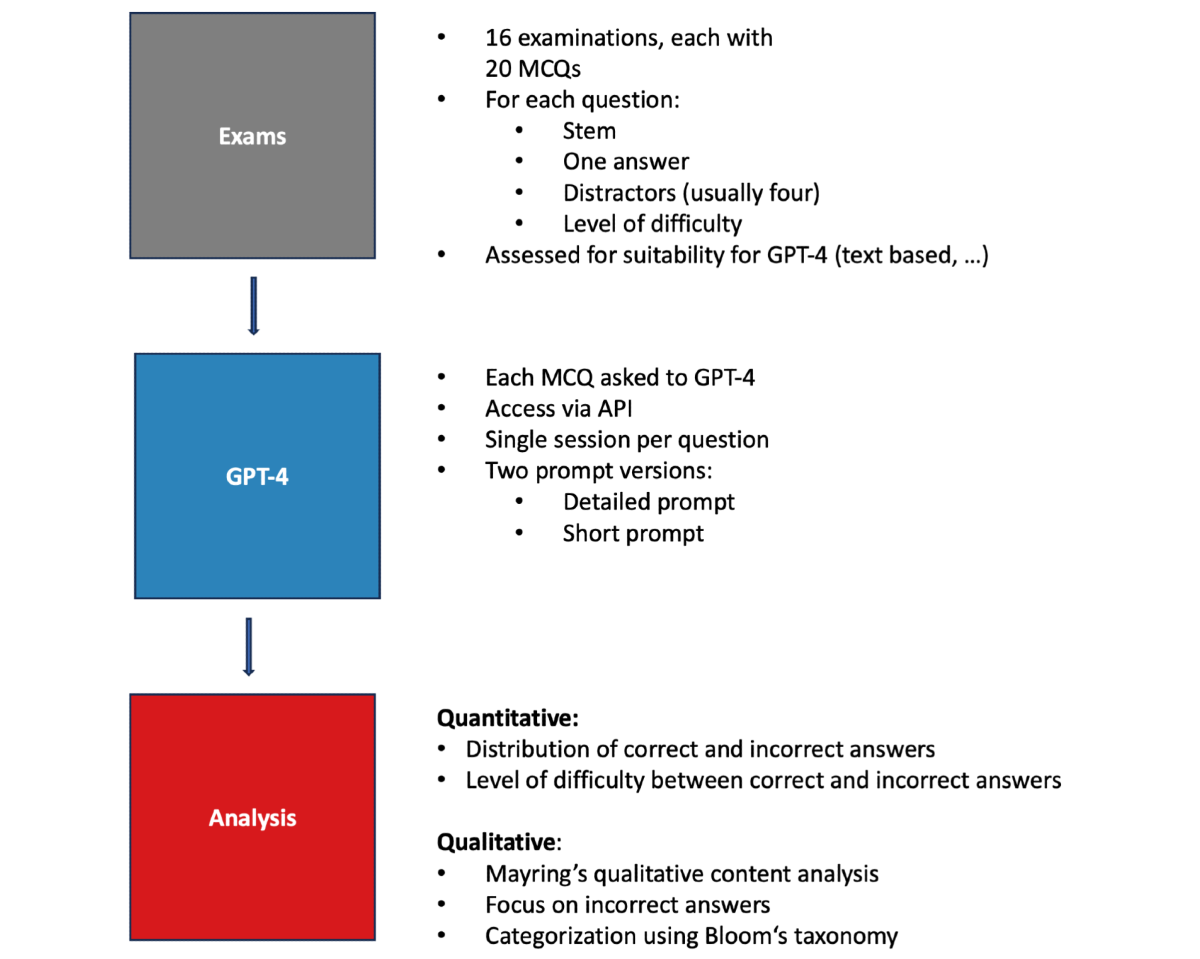
Illustration of our methodological approach. API: application programming interface; GPT-4: Generative Pre-trained Transformer 4; MCQ: multiple-choice question.

A total of 16 examinations from winter term 2014-2015 to summer term 2022 were retrieved from the integrated management system of the Department of Psychosomatic Medicine and Psychotherapy faculty’s web-based exam program (Figure 1 provides a graphical illustration of our methodological approach). In addition to question stems, answers, and distractors, the system also offers quality criteria for each individual question.

Each examination consists of 20 MCQs, with each MCQ having 1 answer and 4 distractors. Diagnostic and therapeutic questions cover topics concerning anxiety disorders, depression, eating, and somatoform and trauma disorders. The examinations also include questions concerning motivational interviewing techniques.

To compare the performance of GPT-4 to the student’s performance, we retrieved the level of difficulty from the system, calculated as the average score attained in the respective question. Undergraduate medical students take the examination containing these questions at the end of their third year, after having attended 7.5 hours of lectures and 18 hours of practical classes on psychosomatic medicine and psychotherapy. To pass, a student is usually required to answer 12 of 20 questions correctly; an adjustment of this passing score is possible if, for example, a question has a too-poor performance.

We assessed the questions for their suitability to be analyzed by GPT-4. From a total of 320 questions, 13 (4%) were excluded, including questions that were not single-choice answers (n=6), ambiguous questions (n=3), questions featuring a graphic that had to be analyzed (n=2), and questions that covered a case represented in multiple questions (n=2).

We used GPT-4 to answer every question (model “gpt-4,” OpenAI LP). For each question, we generated a detailed prompt version and a short prompt version. The prompts were created by the authors in an iterative process using the web interface ChatGPT Plus to achieve ideal performance. The most relevant difference between the versions was that the detailed prompt included a command to critically reflect on the answer and justify the choice made. Table 1 provides an example of a detailed prompt. We used the application programming interface (API) provided by OpenAI to post the questions to GPT-4 and retrieve the answers (access dates were March 21 and March 22, 2023). Each question and answer was posted in its own session. All interactions with GPT-4 were conducted in German, the original language of the examination questions; for the purposes of this paper, the questions were manually translated into English.

**Table 1:** Examples of detailed and short prompt versions.

Prompt version	Example
Detailed prompt	You have all the knowledge of psychosomatic medicine and have to answer an exam question. Please elaborate on the following multiple-choice question. Only one of the five answer choices is correct. Please consider carefully and choose one answer. Give a detailed reason for your answer. At the end of the reasoning, please add the letter of the answer you chose with the following notation: !A! (that is, A between two exclamation marks if you chose answer A). The question reads: A 49-year-old teacher is undergoing rehabilitation treatment after anterior wall infarction. It has been observed that he skips therapy appointments. The wife is concerned because he shows no interest in her visits. When asked, he states that he sees no point in further treatment because he has little hope that his condition will improve. However, none of that really matters anymore. He is also not afraid of another heart attack; on the contrary, then his misery would come to an end. Which suspected diagnosis in the psychosomatic field are you most likely to make? Answer A: Unspecific somatisation disorder; Answer B: Post-traumatic stress disorder; Answer C: Pseudodementia; Answer D: Generalised anxiety disorder; Answer E: Depression.
Short prompt	Please act like a specialist in psychosomatic medicine. Answer the following multiple-choice question and briefly explain your answer: A 49-year-old teacher is undergoing rehabilitation treatment after anterior wall infarction. It has been observed that he skips therapy appointments. The wife is concerned because he shows no interest in her visits. When asked, he states that he sees no point in further treatment because he has little hope that his condition will improve. However, none of that really matters anymore. He is also not afraid of another heart attack; on the contrary, then his misery would come to an end. Which suspected diagnosis in the psychosomatic field are you most likely to make? Answer A: Unspecific somatisation disorder; Answer B: Post-traumatic stress disorder; Answer C: Pseudodementia; Answer D: Generalised anxiety disorder; Answer E: Depression.

### Data Analysis

The responses given by GPT-4 were compared to the answers indicated by the answer index (eg, “A” or “C”) and stored in Excel (version 16.0.10394.20022; Microsoft Corporation).

#### Quantitative Data Analysis

Quantitative analyses and figure generation were performed using R (R version 4.3.1; R Core Team) statistical software [19]. Briefly, we combined all tables with relevant data—that is, answers from GPT-4 and the aggregated data of the students’ exams (such as item difficulty). For each prompt version, we analyzed the ratio of correctly answered questions versus incorrectly answered questions. We further compared the question difficulty (taken from the aggregated student data) across questions answered correctly and incorrectly by GPT-4. The difficulty of a question is operationalized as the proportion of students answering a question correctly, with 0 representing a very difficult question and 1 a very easy question [20]. A Wilcoxon rank sum test was used to test for statistical significance. A level of *P*<.05 was considered statistically significant. If not stated otherwise, the results are given in medians and IQRs.

#### Qualitative Data Analysis

A total of 2 authors (TFW and FH) separately coded each text response. The answers from GPT-4 were analyzed inductively and iteratively according to Mayring’s [21] qualitative content analysis, as described previously by our group [22]. The goal of the analysis was defined in line with the answers to the examination questions. For the main category, we used the correct or incorrect answer to the question, then further focused primarily on incorrect answers.

In the answer texts, individual reasoning was categorized according to Bloom’s taxonomy as revised by Krathwohl [8]. Briefly, we used the following definitions of the cognitive domains for our rating procedure:

1. Remember: retrieving relevant knowledge from long-term memory.
2. Understand: determining the meaning of instructional messages, including oral, written, and graphic communication.
3. Apply: carrying out or using a procedure in a given situation.
4. Analyze: breaking down material into its constituent parts and detecting how the parts relate to 1 another and to an overall structure or purpose.
5. Evaluate: making judgments based on criteria and standards.

In the second step, each of the raters coded the answers using MAXQDA (version 12.3.2; VERBI software). To obtain the same level of abstraction when building the categories, the raters revised the codes together and agreed on the final categories, paraphrasing representative examples and building a hierarchy of categories based on the found codes. Subsequently, both initial raters independently worked through the material again. Each rater individually analyzed the answers given by GPT-4 and built codes using MAXQDA, including the main classification (correct or wrong answer), followed by the category of Bloom’s taxonomy and an example. When they could not agree on a category, 2 other experts were consulted in order to reach a consensus. When GPT-4’s responses were wrong, the explanation was analyzed using the levels of Bloom’s taxonomy (remember, understand, apply, analyze, evaluate, and create) [8].

### Ethical Considerations

The Ethics Committee of the Faculty of Medicine at University Hospital Tübingen approved the study (number 076/2023A). All data were kept anonymous and were not associated with individual students.

## Results

### Quantitative Results

#### Distribution of Correctly and Incorrectly Answered Questions

**Figure 2:**
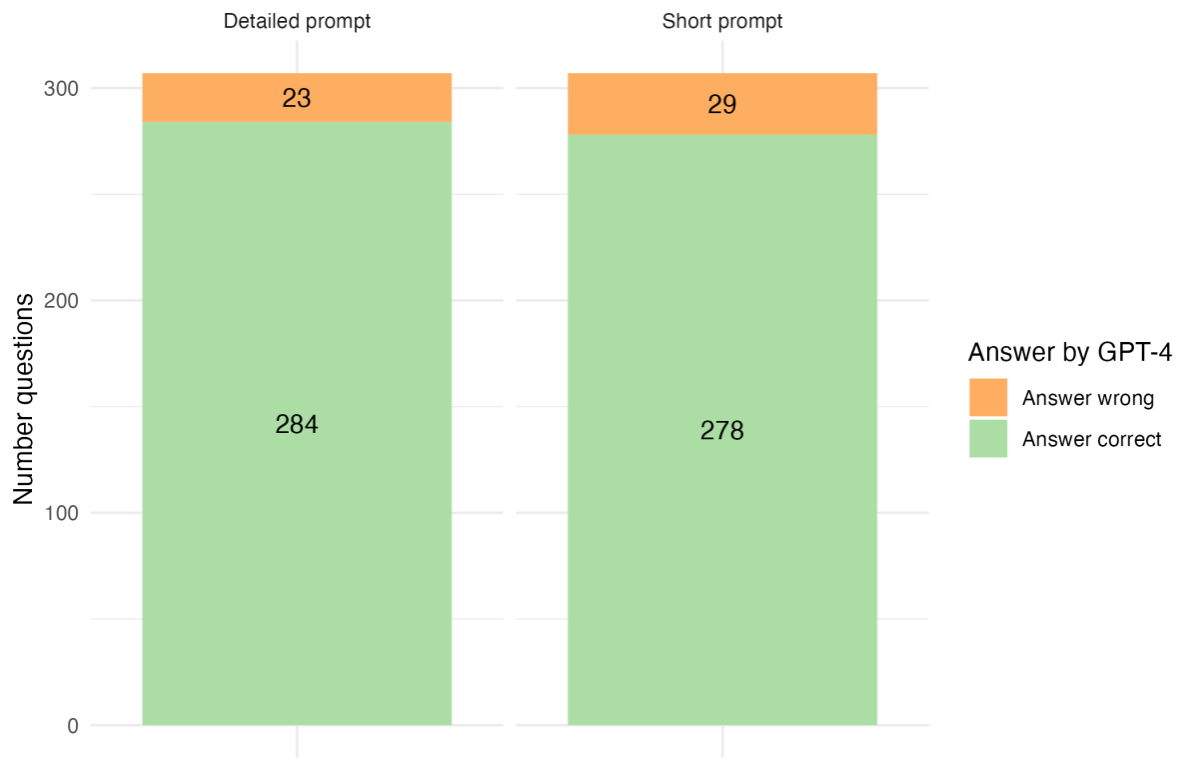
Distribution of correctly and incorrectly answered questions by prompt version.

For the detailed prompt, GPT-4 answered 92.5% (284/307) of the questions correctly; for the short prompt, the success rate was 90.6% (278/307). The distribution is shown in Figure 2.

#### Question Difficulty

**Figure 3:**
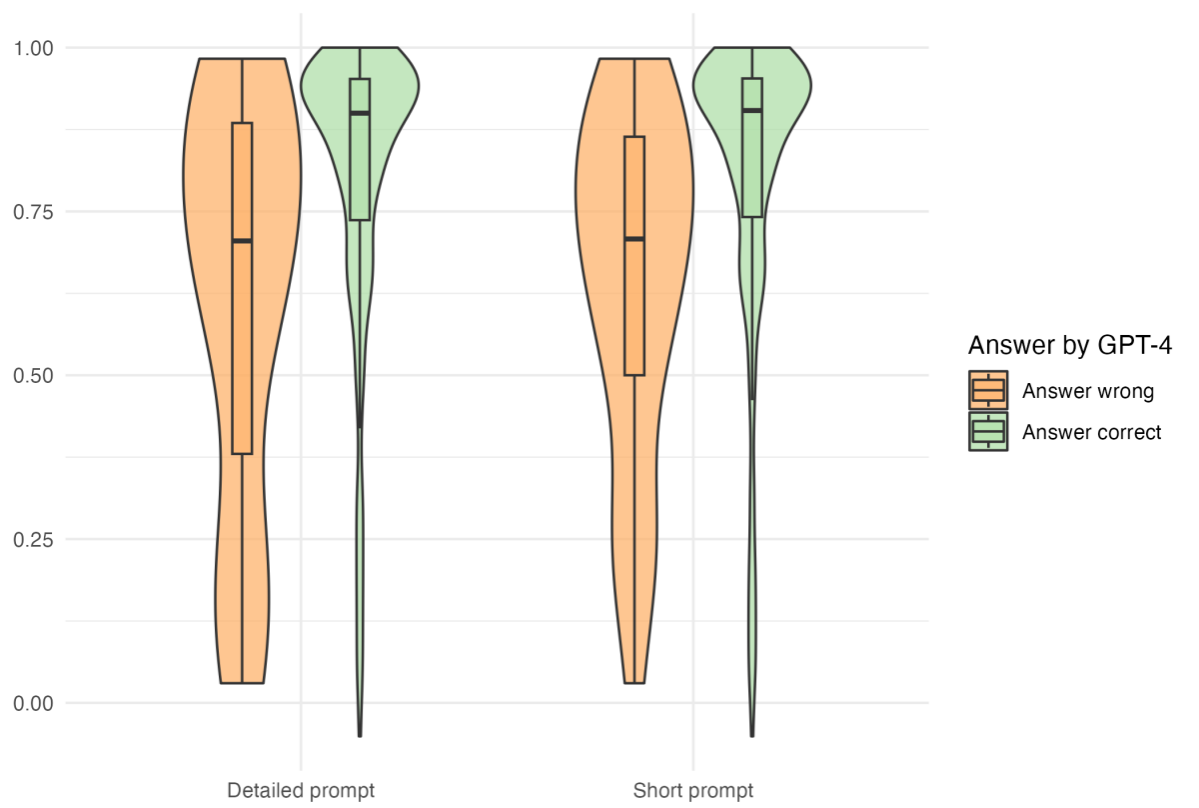
Question difficulty by prompt version and answer correctness.

Across all questions, the median difficulty was 0.892 (IQR 0.705-0.949). The distribution of the question difficulty for correctly and incorrectly answered questions is displayed in Figure 3.

For the detailed prompt, questions answered correctly had a higher difficulty (median 0.900, IQR 0.737-0.952) compared with questions answered incorrectly (median 0.705, IQR 0.380-0.885). This difference was statistically significant (*P*=.002).

In the analysis of the short prompt, we also found a lower difficulty for incorrectly answered questions (median 0.708, IQR 0.500-0.864) compared with correctly answered questions (median 0.904, IQR 0.741-0.953). Here as well, a significant difference was detected between the correctly and incorrectly answered questions (*P*<.001).

#### Exam Scores

**Figure 4:**
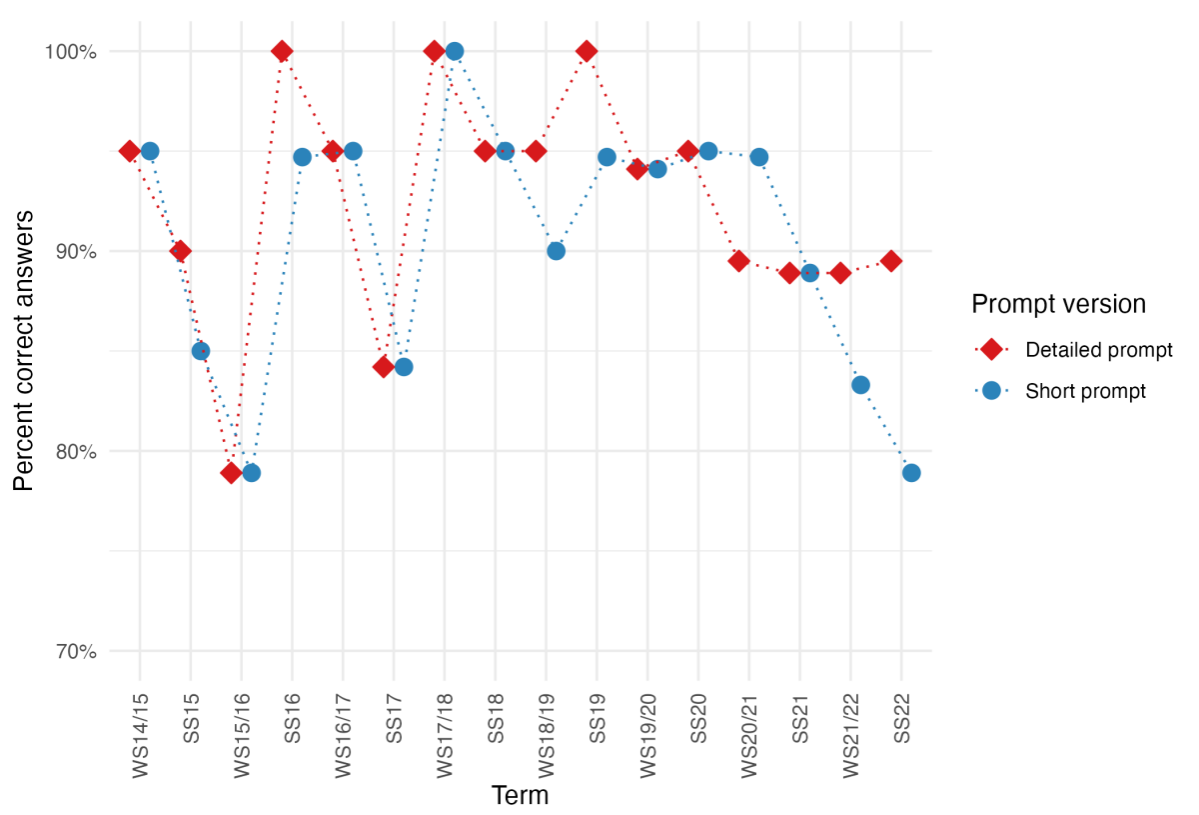
Performance of GPT-4 for all relevant terms for the detailed prompt (red) and short prompt (blue). GPT-4: Generative Pre-trained Transformer 4.

We further analyzed the performance of GPT-4 for all 16 individual exams (Figure 4). Regardless of the prompt version, GPT-4 never scored below 78.9% and thus always passed the exams. Furthermore, 3 exams (exam “WS17/18” for both prompt versions and exams “SS16” and “SS19” for the detailed prompt only) were passed with a score of 100%.

### Qualitative Results

After the quantitative results were obtained, we analyzed the answers given by GPT-4 in detail using content analysis.

#### Correctly Answered Questions

When GPT-4 answered a question correctly, it provided either a detailed or short explanation (Table 2). In the detailed version, GPT-4 also commented on the distractors. For correctly answered questions, the content analysis revealed logical reasoning and the correct use of facts. With regard to the facts cited, no answers were found to be either implausible or incorrect.

**Table 2:** Correct answers by GPT-4 (Generative Pre-trained Transformer 4) to the question described in Table 1 for the detailed and short prompts.

Prompt version	Correct answer
Detailed prompt	Explanation: After careful consideration of the various answer options and taking into account all the knowledge of psychosomatic medicine, answer E: Depression is the most plausible. In this case, the 49-year-old teacher shows clear signs of emotional impairment after an anterior wall infarction. These include disinterest in therapy appointments, lack of interest in his wife’s visits, hopelessness about his recovery, and no sense of fear of another heart attack – on the contrary, he sees the prospect of an end to his “misery” as a positive thing. All these signs point to a possible depressive episode. Answer A: Nonspecific somatization disorder can be ruled out because the patient does not have a multitude of physical symptoms without a demonstrable organic cause. Answer B: Post-traumatic stress disorder could be considered due to the event of the infarction, but the typical symptoms such as memories or flashbacks of the traumatic event, avoidance behaviour, and increased irritability are missing in the description of the case. Answer C: Pseudodementia refers to cognitive impairment caused by depressive symptoms rather than actual dementia. Although this would be possible in the case of depression, the question does not provide enough information about cognitive impairment to support this diagnosis. Answer D: Generalized Anxiety Disorder does not fit the case as the patient states that he is not afraid of having another heart attack and overall does not show excessive fear or anxiety. Therefore, answer E: Depression is the most plausible tentative diagnosis in the psychosomatic field in this case.
Short prompt	Answer E: Depression The symptoms described, such as lack of interest in further treatment, lack of hope for improvement, dejection and even death wishes, most likely indicate depression. The patient shows signs of anhedonia (inability to feel pleasure or interest in activities) and negative thought patterns. In such cases, psychosomatic assessment and appropriate treatment is important to aid the patient’s recovery.

#### Incorrectly Answered Questions

When a question was answered incorrectly by GPT-4, the explanation’s reasoning was analyzed using Bloom’s taxonomy, with its domains “remember,” “understand,” “apply,” “analyze,” “evaluate,” and “create” (Table 3). The most mistakes were found to be related to “remember,” followed by “understand” and “apply,” and only 1 erroneous reasoning example was found for “evaluate.” In the following sections, examples for each category are given in detail.

**Table 3:** Count of incorrect reasonings by GPT-4 (Generative Pre-trained Transformer 4) for each category of Bloom’s taxonomy. For this analysis, all answers (detailed prompts and short prompts) were analyzed together.

Category of Bloom’s taxonomy	Count (n=68), n
Remember	29
Understand	23
Apply	15
Analyze	0
Evaluate	1
Create	0

##### Bloom’s Taxonomy: Remember

This section provides an example of a question for which GPT-4 failed to remember facts. The question presented the case of a man with recurrent physical complaints without organic cause; the complaints persisted for 10 months, making it appropriate for a diagnosis of “undifferentiated somatoform disorder” (F45.1 according to the *International Classification of Diseases, 10th Revision* [ICD-10]). A “somatization disorder” (F45.0) cannot be chosen, as this requires “symptoms of at least 2 years duration” [23].

However, GPT-4 incorrectly chose “somatization disorder.” In its reasoning, GPT-4 stated:

…I would choose answer C: Somatization disorder (F45.0) as the most appropriate diagnosis for this patient. Somatization disorder (F45.0) is characterized by persistent, multiple, and recurrent physical complaints for which no sufficient organic cause can be found. The symptoms can occur in different organ or functional systems and are usually severe enough to interfere with the patient’s daily life.

In its recall, GPT-4 did not mention the required duration of the symptoms stated in the ICD-10. Thus, from what can be observed in the answer, the concept used by GPT-4 missed at least 1 important fact, and this failure could be attributed to GPT-4 not “remembering.”

##### Bloom’s Taxonomy: Understand

An example of GPT-4 not understanding some basic concepts can be found in a question concerning anorexia nervosa. The answer relates to the fact that anorexia nervosa can be accompanied by transient hypercholesterolemia, which does not necessitate treatment [24,25]. However, GPT-4 picked one of the distractors, which states that the basic metabolic rate increases during phases of low body weight and decreases after refeeding [26]—but the opposite is true. In its explanation, GPT-4 thought about these facts (and even remembered them correctly in sentence 3!) but did not seem to understand how they relate and interact.

In patients with anorexia nervosa, the basal metabolic rate is initially elevated at the onset of weight gain and decreases dramatically during refeeding. Food intake should be adjusted accordingly. Basal metabolic rate here refers to the amount of energy the body needs at rest to maintain its basic functions. In people with anorexia nervosa, the body is initially very efficient at obtaining energy due to persistent nutrient deficiency. However, as refeeding progresses, the basal metabolic rate drops dramatically as the body re-learns to store energy.

##### Bloom’s Taxonomy: Apply

The third level of Bloom’s taxonomy involves the application of previously learned content to other areas. An error in this domain was evident when we asked GPT-4 a question about the diagnosis and treatment of a depressive episode. The correct answer was that symptoms or criteria must be present for at least 14 days, which is clearly required by the ICD-10 [23]. However, GPT-4 rates this answer as incorrect and implies that this time frame is more of a rough guideline than a strict minimum requirement.

In the diagnosis of depressive episodes, the time criterion is an important factor. However, many experts believe that a period of at least two weeks is rather a rough guideline. Some patients can also go through a depressive episode in a shorter time or fulfil the criteria for a longer time.

This reveals an issue at the “application” level of Bloom’s taxonomy. GPT-4 seemed to “remember” the fact about the temporal criterion and “understood” its concept. However, it struggled with the “application” of this rule, proposing flexibility where there is conventional rigidity. This can be interpreted as a misapplication of the learned rule and, thus, a mistake made at the application level of Bloom’s taxonomy.

##### Bloom’s Taxonomy: Evaluate

GPT-4 made a mistake at the “evaluate” level of Bloom’s taxonomy in a more complex question testing one’s judgment regarding the ideal therapy for a patient. Due to the complexity, the question is presented here in full:

A 57-year-old ready-to-wear clothing-maker is referred to the psychosomatic outpatient clinic by her family doctor because of persistent back pain. She reports “terrible” pain in her lower back. She has been unable to work for seven months because of it and can hardly sleep. No one was able to help her; the family doctor just sent her from doctor to doctor. (The findings from orthopaedics, neurology, neurosurgery, and radiology that she brought with her show minor disc changes on several levels, but no neurological deficits). She does not want to undergo surgery because “they probably could not earn enough money from her as a patient with public insurance”. Now the family doctor has prescribed her an antidepressant, which she will not take. She still has hope for improvement, but if it continues like this, she does not know whether she will put an end to her life. What is the first course of action indicated?

A: Immediate admission to a psychiatric ward, against the patient’s will if necessary.

B: Initiate inpatient psychosomatic treatment.

C: Observation of the symptoms under medication with NSAIDs.

D: Obtain a neurosurgical second opinion after current imaging.

E: Clarification of secondary disease gain and immediate termination of incapacity for work.

When we used the short prompt, GPT-4 chose the answer B: Plan a psychosomatic inpatient treatment. In its brief explanation, GPT-4 correctly judged why this was the most likely answer.

However, when we used the detailed prompt, GPT-4 chose the distractor E and sent the patient back to work effectively immediately, arguing:

After careful consideration, answer E is most appropriate, but with modification. A detailed explanation of psychosomatic correlations and the possible role of secondary illness gain might help the patient better understand her situation and is thus indicated. However, immediate cessation of incapacity should not be forced, as this could potentially exacerbate the patient’s pain and frustration. It would make sense to first seek outpatient psychosomatic treatment to help the patient cope with her pain and focus on long-term recovery.

In this example, it is evident that GPT-4 knows the facts (remember), understands them (understand), applies them to this situation (apply), and correctly analyzes the situation (analyze). GPT-4 can even be observed to evaluate, but it fails at evaluating the consequences of its decision. While GPT-4 seems to be aware of this and wants to modify the given answer, it incorrectly evaluates the answer options and does not recommend inpatient psychosomatic treatment.

## Discussion

### Principal Findings

Tools based on LLMs are rapidly evolving and influencing medicine [1] and medical education [2]. In this study, we show that GPT-4 yields high accuracy in answering psychosomatic medicine questions, with success rates well above 90%. This confirms results from other studies, which also show high exam scores in, for example, the USMLE (United States Medical Licensing Examination) [14] and other board exams [27]. Although we detected high correct-answer rates for both our detailed prompt and short prompt versions, GPT-4 answered more questions correctly when we used the detailed prompt (284/307, 92.5% vs 278/307, 90.6%). While this study was not designed to assess reasons for this difference, 2 likely explanations for it deserve attention. First, the difference rates for the 2 prompt versions could represent a true difference in the LLM’s performance with respect to the prompt version [28]. Second, the difference could be due to a random variation that is known to occur even when the same prompt is used more than once [29,30].

We were also interested in GPT-4’s performance compared with that of medical students. Here, our analysis revealed that the questions answered correctly by GPT-4 were significantly easier than the questions that were incorrectly answered. This difference could be observed for both the detailed prompt and short prompt versions. For further comparison, it should be noted that question difficulty is not a fixed or static variable but rather is dependent and calculated on the basis of the responses of human students [20].

However, in order to understand why GPT-4 fails at some questions, we further analyzed incorrectly answered questions using a qualitative approach. It is well known that incorrect or inaccurate information is an important issue with LLMs [31]. Bloom’s taxonomy has emerged as a frequently used standard to describe the cognitive process underlying learning [8]. To the best of our knowledge, the levels in Bloom’s taxonomy at which GPT-4 commits cognitive errors have not yet been elucidated. Thus, we performed a detailed assessment of the answers and reasoning provided by GPT-4.

In our analysis, we found that most errors were made at the lowest level of Bloom’s taxonomy, labeled “remember.” In these answers, GPT-4 failed at naming or using a specific fact, as evident in the text response. In the example presented in the previous section, GPT-4 named most of the diagnostic criteria for a somatization disorder but did not mention the time criterion. In this context, it is important to note that GPT-4 has been trained with publicly available and licensed data (although these are not specified in detail by OpenAI) [32]. The information needed in this example is publicly available in the ICD-10 [23] and thus is expected to be included in the GPT-4 training data. Indeed, when asked by the authors, ChatGPT (using GPT-4) states that its training data include details on the ICD-10.

In a recent study, Johnson et al [33] evaluated ChatGPT for its accuracy in providing medical information. Using a quantitative approach, they found that GPT-3.5 provided medical answers ranging from “mostly correct” to “almost correct.” It is important to note that these results were generated using GPT-3.5, an older model than the model we used: GPT-4 is claimed to be “40% more likely to produce factual responses than GPT-3.5” [32] and exhibits better performance in medical exams [27]. However, the results reported by Johnson et al [33] are in line with our findings: while GPT-4 uses most facts correctly and completely, it sometimes fails with respect to specific details. In psychosomatic medicine, we observed this to be a diagnostic criterion; while this can be an issue, missing some specific facts in other areas can make all the difference for patient outcomes. Thus, it is important for those using GPT-4 in medicine to keep in mind that specific facts can be wrong or missing.

Some errors were found at the second level of Bloom’s taxonomy, labeled “understand.” While GPT-4 generally showed good reasoning capability [32] and errors were sparse, we were surprised that some answers yielded obvious logical flaws, as seen in the example from the previous section. In its response, GPT-4 confidently presents a set of sentences that do not correlate logically. Mechanistically, language models such as GPT use likelihood maximization, generating text based on what most likely follows [16,34]. However, this approach can result in what is called hallucinations, or “content that is nonsensical or unfaithful to the provided source content” [34]*.* The resulting medical information might sound very confident but be incorrect [35], thereby posing a significant threat for medical applications [5]. This raises ethical concerns around the use of AI systems for patient-related work, particularly as GPT-4’s algorithms and ethical models are unknown and variable [4]. Because GPT-4 is not considered to be sentient, it neither knows nor cares about the accuracy of its responses.

We also detected some mistakes that represented Bloom’s taxonomy level labeled “application.” In our representative example presented in the previous section, GPT-4 was quite flexible in applying a very strict time criterion. This can be interpreted in the context of the process of training LLMs. Although little has been published about this process, classification systems probably represent only a small amount of the data available on a certain subject. It can be further assumed that information designed for the public might not be as specific as strict diagnostic criteria because it serves another audience. Thus, following GPT-4’s likelihood maximization approach, LLMs might neglect a specific but likely underrepresented piece of information. Furthermore, GPT-4 has been observed to perform poorly in pure calculation tasks [35], probably also challenging strictly numerical criteria. This is not entirely surprising, as GPT-4 is a LLM (with an emphasis on language) and is not intended to be used as a calculator.

We found only 1 mistake that could be classified at the Bloom’s taxonomy level labeled “evaluate,” in which GPT-4 incorrectly judged a medically complex situation.

### Limitations

This study has some limitations that merit discussion. First, we used Bloom’s taxonomy. While it provides good operationalization for cognitive processes, the taxonomy represents a continuum wherein more than 1 level can be activated in a single question [8]. Nevertheless, we observed that most errors could be attributed to only 1 cognitive level. Second, we chose questions from psychosomatic medicine because many aspects of this field can be covered by written language and do not require images or many numbers. However, psychosomatic medicine is a specialty in which treatment can be individualized and especially complex, making categorical judgment harder and possibly reducing generalizability beyond this field. Third, since we only used GPT-4 as our LLM, we cannot judge if our implications hold true for other models. Fourth, our data were acquired 1 time, at a specific date. As the performance of GPT-4 varies over time, this could reduce generalizability [36]. Finally, in this study, we compared GPT-4’s performance with that of medical students by using the difficulty index of each exam question. It is important to note that this difficulty index is specific to the particular cohort of medical students who participated in the representative exam.

### Conclusion

In summary, we found that GPT-4 performs extremely well on psychosomatic medicine questions. Questions answered correctly by GPT-4 were also easier for human students than questions answered incorrectly, as shown by the level of question difficulty. When analyzing the mistakes of GPT-4, we found that most errors corresponded to lower-order cognitive levels, particularly “remember” and “understand.” While we found some mistakes for “apply,” very few or no errors were found for “analyze” and “evaluate” (“create” could not be assessed in this study). To the best of our knowledge, this study is the first to describe the cognitive levels at which GPT-4 makes mistakes in the context of psychosomatic medicine.

This study has important implications. First, GPT-4 is already capable of answering many questions in (psychosomatic) medicine, and thus, should the technology be made available, it could reduce the effectiveness of summative assessment. Second, GPT-4 sometimes fails at exact facts, correct understanding, and application of knowledge; however, without exact knowledge, these failures are hard to recognize. Thus, the output generated by GPT-4 must be checked for accuracy, especially in those domains. Our research can also help in model training, and future studies can use our results to correlate model training and LLM outcomes.

## Abbreviations

AI: artificial intelligence
API: application programming interface
GPT: Generative Pre-trained Transformer
ICD-10: International Classification of Diseases, 10th Revision
LLM: large language model
MCQ: multiple-choice question
USMLE: United States Medical Licensing Examination

## Data Availability

The data sets used and/or analyzed during this study are available from the corresponding author upon reasonable request.
